# *Notes from the Field:* Multistate Outbreak of *Salmonella enterica* I 4:I:- Infections Linked to Charcuterie-Style Meats — United States, 2023–2024

**DOI:** 10.15585/mmwr.mm7417a2

**Published:** 2025-05-15

**Authors:** Angelo Lodato, Andrea Cote, Joshua Rounds, Laurie K. Stewart, Anna E. Pickett, Megan E. Cahill, Lorrie L. B. Martin, Jennifer K. Adams, Lauren Gollarza, Zachary D. McCormic

**Affiliations:** ^1^Oak Ridge Institute for Science and Education, Oak Ridge, Tennessee; ^2^Division of Foodborne, Waterborne, and Environmental Diseases, National Center for Emerging and Zoonotic Infectious Diseases, CDC; ^3^Office of Public Health Science, Food Safety and Inspection Service, U.S. Department of Agriculture, Washington DC; ^4^Minnesota Department of Health; ^5^Washington State Department of Health; ^6^Vermont Department of Health; ^7^Career Epidemiology Field Officer Program, CDC; ^8^Association of Public Health Laboratories, Bethesda, Maryland.

**Summary**

**What is already known about this topic?**

Three multistate U.S. outbreaks of salmonellosis linked to charcuterie-style meats have been reported since 2010.

**What is added by this report?**

During December 2023–March 2024, CDC and public health partners in 33 states investigated an outbreak of *Salmonella* *enterica* I 4:I:- infection linked to contaminated charcuterie-style meats. Early usage of purchase records identified a common charcuterie product from one company and enabled a swift recall.

**What are the implications for public health practice?**

Charcuterie-style or fermented, salt-cured, or dried meats are susceptible to bacterial contamination from underprocessing. The U.S. Department of Agriculture Food Safety and Inspection Service has recommendations for facilities for the safe production of charcuterie-style products. Consumer purchase records are an important tool in foodborne outbreak investigations and can lead to prompt regulatory action.

On December 19, 2023, CDC’s PulseNet, the national molecular subtyping network for foodborne disease surveillance, identified a cluster of 13 *Salmonella enterica* I 4:I:- infections in five states that were found to be highly related by whole genome sequencing, indicating a possible outbreak.* CDC, the U.S. Department of Agriculture Food Safety and Inspection Service (USDA-FSIS), and state and local partners conducted an investigation to identify the outbreak source. Patients’ self-reports of foods consumed before illness onset identified similar reports of charcuterie products, and clinical isolates were genetically related to a 2021 outbreak linked to salami sticks.^†^

## Investigation and Outcomes

An outbreak-associated case was defined as a *Salmonella* I 4:I:- infection in a person with an isolate related within three allele differences to the outbreak strain (using core genome multilocus sequence typing), with illness onset between November 20, 2023 and February 10, 2024 (Figure). Overall, PulseNet detected 104 cases across 33 states. The median patient age was 48 years (range = 1–92 years); 40% of patients were female. Twenty-seven patients were hospitalized^§^; none died.

**FIGURE:**
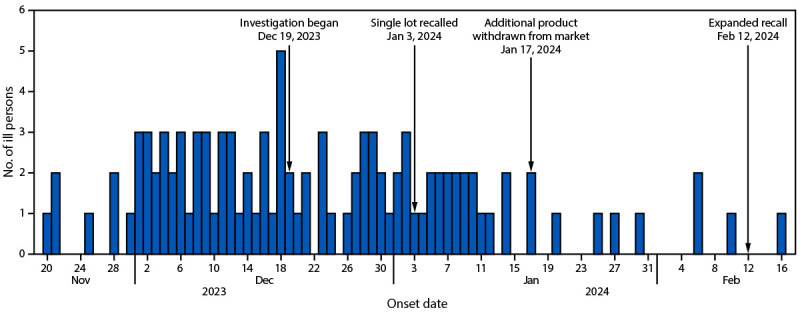
Number of ill persons infected with *Salmonella enterica* I 4:I:-, by illness onset date* — United States, 2023–2024 * If illness onset date was missing, specimen collection date minus 3 days was plotted as a proxy for onset date.

During the initial interview with state health partners, two patients reported having consumed the same company A charcuterie product, which contained three ready-to-eat (RTE) fermented, salt-cured, and dried pork products (coppa, salami, and prosciutto) during the week preceding illness. Both patients purchased the product from different locations of the same national grocery chain. Three days after the outbreak was identified, CDC provided a questionnaire to states to collect more information on charcuterie product exposures and requested purchase records from the grocery store chain (e.g., receipts and shopper card histories) for patients who shopped at the common grocery chain and consented to record collection. This activity was reviewed by CDC, deemed not research, and was conducted consistent with applicable federal law and CDC policy.^¶^

Among 68 patients from 27 affected states with food history information available, 50 (74%) reported consuming charcuterie-style meats before illness onset, a percentage statistically significantly higher than that reported in the 2018–2019 FoodNet Population Survey used to identify when consumption of a food is elevated in an outbreak (39%; p<0.001 using a binomial probability distribution).**

The Minnesota Department of Agriculture isolated the outbreak strain from a company A charcuterie product from a patient’s home, prompting a recall of that specific product lot. *Salmonella* was not detected in company A charcuterie products tested by Washington and Vermont public health partners. Sixteen consumer purchase records, representing purchases from 10 states, were shared with USDA-FSIS, which conducted a traceback investigation. The purchase records identified 11 charcuterie purchases, representing multiple product lots, that traced back to two of company A’s federally regulated production facilities. The remaining purchases traced back to different suppliers.

USDA-FSIS conducted for-cause food safety assessments (FSA) (i.e., a comprehensive review of an establishments’ food safety system because of a specific reason, including a foodborne illness outbreak) at the two company A production facilities and collected 179 product, food contact, and environmental samples (*1*). Two samples of an RTE product that contained coppa yielded two nonoutbreak strains of *Salmonella*. FSAs conducted in company A’s production facilities identified the potential for inadequate salt-curing and drying (underprocessing) of coppa during production, which could result in the presence of *Salmonella* species in the finished product. USDA-FSIS has a zero-tolerance standard for *Salmonella* contamination in RTE products. The combination of the positive *Salmonella* results from RTE products, epidemiologic information, and potential for underprocessing of coppa observed during FSAs led to an expanded recall of all products from company A containing coppa that were still within shelf life on February 12.

## Preliminary Conclusions and Actions

This report describes the largest U.S. infectious outbreak associated with charcuterie-style meats since 2010.^††^ Charcuterie-style meats are typically produced using either fermentation and drying or salt-curing and drying rather than cooking. Although the production process should control bacteria that can cause illness in consumers, if the procedures are not followed carefully, charcuterie-style meats could be underprocessed and presence of bacteria poorly controlled. Facilities are required by USDA-FSIS to demonstrate sufficient scientific evidence that production processes for charcuterie-style meats will result in a 5-log reduction of Salmonella or an alternative pathogen lethality and will achieve shelf-stability among other controls^§§^ (*2*).

Consumer purchase records were successfully used for hypothesis generation in this outbreak investigation and provided a critical foundation for traceback activities. Coupled with patient interview information, the use of purchase records enabled rapid identification and recall of contaminated products 17 days after the investigation began (approximately 1 week faster than previous CDC-led foodborne investigations that did not use purchase records), likely preventing additional cases of illness. Purchase records are important tools supporting epidemiologic and traceback efforts and can facilitate implementation of prompt regulatory action.

## Footnotes

*
Outbreak Detection | PulseNet | CDC

†
Salmonella Outbreak Linked to Salami Sticks | CDC

§ Information about whether patients are or are not hospitalized is collected as part of foodborne illness outbreak investigations; however, information on clinical course of hospitalized patients is not collected.

¶ 45 C.F.R. part 46, 21 C.F.R. part 56; 42 U.S.C. Sect. 241(d); 5 U.S.C. Sect. 552a; 44 U.S.C. Sect. 3501 et seq.

**
FoodNet Fast | CDC

††
BEAM Dashboard | CDC

§§
9 CFR 417 - HAZARD ANALYSIS AND CRITICAL CONTROL POINT (HACCP) SYSTEMS - Content Details - CFR-2024-title9-vol2-part417

## References

[1] US Department of Agriculture, Food Safety and Inspection Service. Food safety assessment methodology – revision 6. Washington, DC: US Department of Agriculture; 2024. https://www.fsis.usda.gov/policy/fsis-directives/5100.1

[2] US Department of Agriculture, Food Safety and Inspection Service. FSIS ready-to-eat fermented, salt-cured and dried products guideline. Washington, DC: US Department of Agriculture; 2023. https://www.fsis.usda.gov/guidelines/2023-0002

